# Mutational analysis of cytocentrifugation supernatant fluid from pancreatic solid mass lesions

**DOI:** 10.1002/dc.23048

**Published:** 2013-11-22

**Authors:** Sydney D Finkelstein, Marluce Bibbo, Thomas E Kowalski, David E Loren, Ali A Siddiqui, Charalambos Solomides, Eric Ellsworth, Dara Aisner, Anders Hjerpe

**Affiliations:** 1 RedPath Integrated Pathology, Inc.Pittsburgh, Pennsylvania, USA; 2 Division of Gastroenterology and Hepatology, Department of Internal Medicine, Thomas Jefferson University HospitalPhiladelphia, Pennsylvania, USA

**Keywords:** pancreas, cytology supernatant, mutational analysis

## Abstract

Diagnosis of fine-needle aspirations of pancreatic solid masses is complicated by many factors that keep its false-negative rate high. Our novel approach analyzes cell-free cytocentrifugation supernatant, currently a discarded portion of the specimen. Supernatant and cytology slides were collected from 25 patients: 11 cases with confirmed outcome [five positive (adenocarcinoma) and six negative (inflammatory states)], plus 14 without confirmed outcomes. Slides were microdissected, DNA was extracted from microdissections and corresponding supernatants, and all were analyzed for KRAS point mutation and loss of heterozygosity. Notably, higher levels of free DNA were found in supernatants than in corresponding microdissected cells. Supernatants contained sufficient DNA for mutational profiling even when samples contained few to no cells. Mutations were present in 5/5 malignancies and no mutations were present in inflammatory states. In conclusion, these findings support using supernatant for mutational genotyping when diagnostic confirmation is required for pancreatic solid masses. Diagn. Cytopathol. 2014;42:719–725. © 2013 Wiley Periodicals, Inc.

Advances in cross-sectional and endoscopic imaging, coupled with improvements in fine-needle aspiration (FNA) sampling technology, have led to an increase in the number of specimens for cytology evaluation.^1^–^9^ This is true for many organ systems and can be exemplified by the pancreas where the number of solid masses, cysts, and ductal brushing specimens for cytology examination continues to rise.^10^,^11^ These advances have also allowed for smaller lesion sizes to be detected at first diagnosis, resulting in earlier detection and treatment of cancer and precancerous processes. At the same time, enhanced detection and sampling techniques have lead to a commensurate increase in the number of benign lesions and mimics of neoplasia that otherwise would remain undetected in asymptomatic patients. All this requires the cytologist to achieve definitive diagnosis with ever-smaller specimens. Advances in detection must be coupled with equally reliable results of diagnosis, as indeterminate diagnostic results will limit effective patient management despite better imaging and sampling methodology.^12^–^16^

Besides limitations in sample quantity, another hurdle in cytology practice is sampling variation, given that neoplastic processes often are topographically heterogeneous across a particular organ or tissue. This heterogeneity operates at both a cellular and a molecular level of analysis and must be considered whenever sampling from within a larger sized lesion is undertaken.^17^ Recently, directed cyst wall biopsy has become available for the work up of cystic lesions of the pancreas.^18^ Pancreatic cysts are especially challenging since the aspirated fluid is collected from the center of the cyst while critical cellular events are likely taking place focally along the lining at the periphery of the cyst and associated duct passages. Biopsy of the cyst wall can be most helpful in accessing the lining cells, however sampling variation is still possible as the cyst/dilated duct lining is not likely to be uniformly altered over its full extent.

Furthermore, depending upon the precise location of biopsy or cytobrush sampling, sites representing the most advanced disease progression may be missed, thereby reducing detection sensitivity of advanced dysplasia or cancer. The ability to evaluate cellular and molecular markers of neoplastic change over a wider distance than what is confined to the sampling site would be beneficial because the impact of sampling variation could be reduced. In this way, ancillary tools that utilize markers to detect cancer-associated change could significantly improve the detection and characterization of neoplasia. One of the most important challenges in the clinical application of molecular discovery is to find the best markers and methods of specimen handling that complement existing cytology practice without competing for cellular specimens needed for comprehensive microscopic analysis.^17^

Complementary approaches do exist, the most notable being the microdissection of cells from unstained recut cell block tissue sections or stained cytology slides guided by microscopic features.^19^,^20^ Relying on polymerase chain reaction (PCR) to amplify small amounts of representative DNA, microdissection of FFPE and cytology slides is a well recognized technique that can resolve indeterminate microscopic diagnosis as well as provide clinically actionable information not otherwise obtained by microscopic cellular examination.^21^,^22^ In particular the clinical utility of microdissection-based molecular analysis for loss of heterozygosity (LOH) and KRAS has been demonstrated in pancreaticobiliary disease, with LOH and KRAS point mutations universally recognized as hallmarks of pancreatic cancer.^23^–^28^ A drawback of the microdissection approach is the requirement for adequate number of representative cells isolated from non-neoplastic support cells. When specimen cellularity is low, there can be a reluctance to utilize limited numbers of stained cytology cells for molecular analysis, especially when the most representative cells are confined to a single glass slide.

Because neoplastic processes are fundamentally characterized by increased cell turnover and progressive mutation acquisition, it is reasonable to hold that the local cellular environment, regional lymphatic drainage, and systemic circulation may contain DNA emanating from a malignancy. Such DNA may be used to test for the presence of cancer, and work has shown that tumor DNA and RNA can be detected in microscopically negative lymph nodes as well as the circulation.^29^–^31^ PCR-based methods can be employed on cytology specimens, such as fine-needle aspirates, to interrogate the cells themselves as well as any free DNA that is included in the aspirated samples. The DNA present in supernatant fluid that is left after cytocentrifugation for cytology preparation may be indicative of carcinogenic changes, given the presence of cancer-related mutations that can be found in such fluid of specimens taken from other sites (i.e., the biliary tract).^32^ Here, fine-needle-aspirated cytology samples of pancreatic solid and cystic masses containing cellular and free DNA were interrogated to yield information as to the presence or absence of cancer-associated molecular changes.

## Methods

### Study Population

After receiving appropriate IRB approval, stained cytology slides and corresponding supernatant fluid specimens were collected from 25 patients. Cytology adequacy assessment and diagnosis were carried out using recognized morphologic criteria.^33^ The standard cytology procedure for fine-needle aspiration biopsies samples involved mixing the fine-needle aspirate with 5–10 ml of Saccomanno’s fixative after which the sample was centrifuged and cells pelleted onto glass slides. The residual supernatant, usually maintained for several weeks at 4°C and then discarded, was used for DNA extraction and mutational analysis.

Outcomes were established for 11 specimens, with five confirmed positive (pancreatic adenocarcinoma) and six negative (*n* = 6) pancreatic solid mass specimens. Both positive and negative outcomes were confirmed by surgical pathology. The remaining 14 patients had pancreatic solid masses or pancreatic cyst wall biopsy samples of cystic lesions without known outcomes, and were included for comparison between the supernatant and microdissected cytology slides as specimens for mutational profiling.

### Molecular Analysis

Mutational profiling was performed on the microdissected stained cytology and the findings compared to extracted DNA from the corresponding cytocentrifugation supernatant fluid. Microdissection of stained cytology slides was carried out as previously described.^16^,^23^–^27^ Supernatant fluid (2 ml) underwent DNA extraction (Qiagen, Valencia, CA). The resulting DNA was resuspended in a small volume of hypotonic buffer and quantified by optical density (NanoDrop, Thermo Scientific, Wilmington, DE). Microdissected stained cytology cells underwent equivalent DNA extraction and resuspension. DNA amplifiability was determined by quantitative PCR targeting a 150 base pair length of the first coding exon of the KRAS oncogene. KRAS point mutation determination targeted codon 12 and 13 using dideoxy chain termination as previously described.^16^,^23^–^27^ Allelic imbalance was assessed for LOH with a panel of 16 microsatellite markers targeting common sites for tumor suppressor genes associated with pancreaticobiliary cancer at the following chromosomal locations: 1p (CMM1, Lmyc), 3p (VHL, OGG1), 5q (MCC, APC), 9p (CDKN2A, CDKN2B), 10q (PTEN, MXI1), 17p (TP53), 17q (NME1, RNF34), and 21q, 22q (NF2) using quantitative fluorescent PCR/capillary electrophoresis.^16^,^23^–^27^ The marker panel has previously undergone analytic and clinical validation for pancreaticobiliary disease as reported in prior studies.^16^,^23^–^27^

Quantitative allelic imbalance determination was performed as previously described.^34^ In short, the threshold for significant allelic imbalance for each microsatellite marker of the LOH marker panel was based on a large database of non-neoplastic aspirated pancreatic cyst fluid and microdissected stained cytology samples with confirmed outcome by surgical pathology and clinical follow up. This large dataset of over 1,000 specimens encompassed the majority of allelic combinations seen in the general patient population available both as unfixed and fixative-treated extracted DNA. The range for normal allelic balance was defined as two standard deviations from the average allelic ratio in which the fluorescence derived from the shorter allele copy is divided by that of the longer allele copy.^34^ Allelic ratios falling outside the thresholds were considered as demonstrating significant imbalance (LOH). When imbalance was shown to be present, an LOH clonality (degree of clonal expansion) measurement was approximated using the formula 1-[(sample allelic copy ratio)/(average allele copy ratio for allele pairing)] × 100% when the shorter microsatellite allele copy was found to be relatively deficient. The inverse of this formula was applied when the longer allele copy was relatively deficient. For KRAS point mutation assessed by dideoxy chain termination, the ratio of wild type and mutant peak heights was used as an approximation of mutated versus non-neoplastic DNA for an individual sample. It is recognized that clonality determination for oncogene point mutation and allelic imbalance is at best an approximation as the fluorescence output by capillary electrophoresis is not necessarily stoichiometric but representative for the allelic pairing ratio of an individual patient sample.^35^,^36^

## Results

A total of 25 cytocentrifugation supernatant and microdissected cytology pancreatic fine-needle aspiration specimens were analyzed for DNA content and mutational profiling and were compared to their respective cytology findings (Tables 1 and 2). Eleven of these cases were from direct fine-needle aspirations of solid pancreatic mass lesions with confirmed outcome. Each case first received a cytology assessment pertaining to adequacy for microscopic evaluation. Molecular analysis was carried out separately on DNA extracted from microdissected cells judged to be most representative of the cytologic diagnosis, reflecting the greatest degree of cellular atypia present in an individual sample.

**Table 1:** Correlative Analysis of DNA Quantity and Mutational Analysis Between Microdissected Stained Cytology and Corresponding Cytocentrifugation Supernatant Fluid

Pt	Microdissected cytology cells		Cytocentrifugation supernatant		Increased DNA yield (ng/µL)	Comparison of mutations
	DNA quantity (ng/µL)	KRAS/LOH mutations (clonality)	DNA quantity (ng/µL)	KRAS/LOH mutations (clonality)		
1	4.2	LOH: 9p (low)	15.4	LOH: 9p (low)	11.2	Equivalent
2	1.8	KRAS: 12R (low) LOH: 1p (low), 22q (low)	14.7	KRAS: 12R (low) LOH: 1p (low), 22q (high)	12.9	Higher mutation clonality
3	7.5	KRAS 12V (high) LOH: 9p (low), 17p (low)	11.7	KRAS 12V (high) LOH: 9p (high), 17p (high)	4.2	Higher mutation clonality
4	6.4	KRAS: 12V (low)	13.1	KRAS: 12V (low) LOH: 1p (low), 3p (low), 9p (low), 17p (low)	6.7	Additional mutations detected
5	8.5	KRAS: 12R (low)	64.1	KRAS: 12R (high): LOH: 5q (low), 10q (high), 17q (high)	55.6	Higher mutation clonality and additional mutations detected
6	2.2	No mutations	6.2	No mutations	4.0	Equivalent
7	1.8	No mutations	4.1	No mutations	2.3	Equivalent
8	3.4	No mutations	4.7	No mutations	1.3	Equivalent
9	1.1	No mutations	9.3	No mutations	8.2	Equivalent
10	1.4	DNA did not amplify	3.3	No mutations	1.9	Equivalent
11	0.9	DNA did not amplify	3.2	No mutations	2.3	Equivalent
12	3.3	No mutation	14.5	KRAS: 12G (low)	11.2	Additional mutations detected
13	Not tested		8.9	No mutation	Not applicable	
14	0.8	No mutation	15.7	No mutation	14.9	Equivalent
15	5.8	LOH: 9p (low)	26.8	LOH: 9p (low)	21.0	Equivalent
16	Not tested		6.2	No mutation	Not applicable	
17	2.4	KRAS: 12D (low)	8.8	KRAS: 12D (high)	6.4	Higher mutation clonality
18	1.3	DNA did not amplify	7.7	KRAS: 12V (low), KRAS: 12R (low)	6.4	Additional mutations detected
19	Not tested		4.1	No mutation	Not applicable	
20	2.6	No mutation	22.1	No mutation	19.5	Equivalent
21	8.4	DNA did not amplify	32.8	KRAS: 12V (low), KRAS: 12R (low)	24.4	Additional mutations detected
22	5.1	No mutation	15.5	LOH: 10q (low)	10.4	Additional mutations detected
23	2.8	No mutation	11.8	LOH: 17p (low)	9.0	Additional mutations detected
24	4.3	DNA did not amplify	17.7	KRAS: 12D (low)	13.4	Additional mutations detected
25	12.7	DNA did not amplify	46.2	KRAS: 12D (high)	33.5	Additional mutations detected

These specimens represent pancreatic fine-needle aspirates of pancreatic mass and cyst wall lesions, and the data shown to compare information available from two parts of the sample, microdissected stained cytology cells and cytocentrifugation supernatant fluid.

**Table 2:** Correlative Cytologic and Molecular Features of Positive and Negative Solid Pancreatic Mass Control Samples

Pt	Outcome from surgical pathology or cytology	Cytology	Microdissected cytology cells		Cytocentrifugation supernatant	
		Degree of cellularity	Diagnosis	KRAS/LOH mutations	Diagnosis	KRAS/LOH mutations
1	Adenocarcinoma	High	+	LOH: 1 low clonality	+	LOH: 1 low clonality
2	Adenocarcinoma	High	+	KRAS: low clonality mutation LOH: 2 low clonality	+	KRAS: low clonality LOH: 1 low clonality, 1 high clonality
3	Adenocarcinoma	Moderate	+	KRAS: high clonality LOH: 2 low clonality	+	KRAS: high clonality LOH: 2 high clonality
4	Adenocarcinoma	Low	+	KRAS: low clonality	+	KRAS: low clonality LOH: 4 low clonality
5	Adenocarcinoma	Low	+	KRAS: low clonality	+	KRAS: high clonality LOH: 2 high clonality, 1 low clonality
6	Pancreatitis	Moderate	−	No mutations	−	No mutations
7	Pancreatitis	Moderate	−	No mutations	−	No mutations
8	Pancreatitis	Low	−	No mutations	−	No mutations
9	Pancreatitis	Low	−	No mutations	−	No mutations
10	Pancreatitis	Acellular	NR	DNA did not amplify	−	No mutations
11	Pancreatitis	Acellular	NR	DNA did not amplify	−	No mutations

These solid mass pancreatic fine-needle aspirates had confirmed diagnosis based on cytology and/or surgical pathology. Mutational profiling results are shown for positively detected mutation. KRAS point mutations denote codon 12 amino acid substitutions. Mutations were classified as low clonality, when the mutation was present in less than 75% of the DNA, or high clonality, when the mutation was present in greater than 75% of the DNA (NR = no result; + = malignant; − = benign malignancy detected).

DNA levels were compared between the microdissection cytology and cytocentrifugation supernatant fluid specimens to establish a sense of the relative amount DNA obtainable from each type of specimen. In every case in this series, a greater amount of DNA was obtained from the supernatant fluid than that extracted from microdissected stained cytology cells, an average of 15.5 ng/µL for the former compared to an average of 4.0 ng/µL for the latter (Table 1). Quantitative PCR analyses showed that the amount of amplifiable DNA was equal or greater in the supernatant specimens (data not shown). Importantly, all DNA samples obtained from the supernatant were amplifiable.

The DNA concentration values reported here should not be regarded as precisely comparable measurements of the specimen, as only a portion of the supernatant fluid and stained cytology cells underwent extraction of DNA. Furthermore, in vitro DNA degradation effects related to fixative exposure and staining could be responsible, in part, for diminished amplifiability of microdissected stained cytology cells. Nonetheless, the supernatant fluid still yielded distinctly higher amounts and more intact, amplifiable DNA compared to microdissected cells.

Comparing the two sources of DNA, microdissected stained cytology cells and cytocentrifuged supernatant, detectable mutational change was equal or greater in the supernatant specimens. In all cases, mutational clonality was equal to or higher in the supernatant DNA compared to the microdissected cell DNA, further supporting the concept that supernatant contains DNA from neoplastic cells in patients with confirmed malignancy (Tables 1 and 2). Because mutation detection in FNA specimens entails analysis of a combination of representative lesional cells, potentially neoplastic in origin, admixed with contaminating non-neoplastic supporting cells, higher clonality in the supernatant samples supports enrichment of neoplastic cell DNA in the extracellular space component of the FNA specimen as compared to microdissected cytology cells.

The ability of mutational analysis to detect mutational change was assessed using positive and negative controls confirmed by surgical pathology (Table 2). Mutational analysis of either microdissected cytology slides or supernatant fluid revealed no malignant samples that were false negative, and no benign samples that were false positive. Therefore, the performance characteristics of both microdissected cytology slides and supernatant fluid were 100% specific and 100% sensitive for malignancy. Six cases (patients 4, 5, and 8–11) contained few to no cells for diagnosis. However, four of those cases (patients 4, 5, 8, and 9) were still correctly diagnosed through molecular methods - two of these four cases were diagnosed only after analyzing the supernatant.

## Discussion

According to the National Cancer Institute, over 45,000 Americans will be diagnosed with pancreatic cancer in 2013, and although FNA samples remain the common practice for diagnosing pancreatic cancer, analysis of FNA samples is not always conclusive and a substantial number of false-negative diagnoses occur.^37^–^40^ Diagnosing pancreatic cancer using FNA samples is hampered by many factors including variation in the quantity and/or quality of samples as well as indeterminate cytologic characteristics.^38^–^40^ Any of these can decrease the chances of obtaining a definitive diagnosis for a patient.

Using a small group of pancreatic FNA specimens, this study demonstrated that the cytocentrifuged supernatant component of the specimens contained abundant cell-free DNA to generate mutational profiles that closely matched the corresponding profiles of cells present, and was, in some cases, a better source for DNA and subsequent mutational detection than cells on microdissected stained cytology slides (Table 1). The molecular panel used to examine both microdissected stained cytology slides and supernatant fluids confirmed the presence of cancer-associated changes in specimens with morphologically malignant surgical pathology or cytology outcomes. No mutations were present in cases of inflammation. Both microdissected stained cytology slides and supernatant fluid molecular analysis provided diagnoses in cases where cytology was low or acellular (Table 2). Particularly of note, the supernatant fluid was able to provide diagnoses in two instances in which the other methods could not.

Because supernatant fluid is typically discarded during preparation of cells for cytology, analysis of supernatant fluid affords an additional way to characterize molecular changes, contributing valuable information as to the presence of neoplastic cell proliferation, especially when cytology results are unclear or acellular. When additional discriminating information is needed beyond cytology, this investigation demonstrated that the supernatant fluid can be utilized as a source of molecular information that could become a powerful complement to standard cytology evaluation. In fact, the interrogation of free DNA for cancer applications is not without precedent. Investigations of DNA in serum and plasma have shown that it may be more representative of a tumor than intracellular DNA obtained by various methods.^33^,^41^,^42^

While all of the supernatant specimens evaluated here did provide adequate DNA for mutational profiling, it is expected that, in practice, a small proportion of markedly hypocellular specimens will not meet the lower quantity of DNA required for mutational analysis. In light of the present findings that mutation-bearing free DNA is often present in hypocellular specimens with neoplasia, we speculate that hypocellular specimens that lack sufficient DNA would be from non-neoplastic states, given the lack of rapidly replicating cells. However, this concept requires confirmation through additional testing. Studies are now underway to further test the clinical actionability of the molecular information from cytocentrifugation specimens.

Several limitations of this molecular analysis of cytocentrifugation supernatant are recognized. The total number of test samples was not large, and the promising results shown here need to be evaluated with a greater number of specimens. In addition, this study was restricted to the use of Saccomanno’s fixation. Ideally, each commonly used fixative should be individually tested for its capacity to deliver adequate levels of representative supernatant DNA for mutational profiling. It is reasonable, however, to expect favorable results with other methods of sample preparation since most cytology fixatives are alcohol based and are not expected to induce significant DNA degradation. Consistently, prior work has shown that cytology specimens based on microdissected stained cytology cells, are especially suitable for mutational analysis.^22^,^24^,^25^,^27^

One of the greatest challenges to the early diagnosis of cancer is sampling variation due to the topographic tissue heterogeneity that is so often seen in solid organ neoplasia. This is particularly true for pancreatic masses, where neoplastic disease can be missed if the aspiration does not capture the most advanced neoplastic cells. Sampling variation can occur when the most advanced stage of neoplastic disease development is missed or when inflammation or stromal cells are included during the aspiration process. In many cases, it may not be possible to discriminate between neoplastic and non-neoplastic epithelial cells by microscopy alone.^43^–^45^

Data from the supernatant fluids of FNA cytology specimens illustrate that the supernatant consistently provided ample, amplifiable DNA for mutational detection, even when the respective cytology sample lacked sufficient cellularity. Moreover, in many cases, supernatant DNA offered enhanced mutational detection when compared to what cytology or microdissected stained cytology cells could supply, supporting the theory that supernatant is enriched with neoplastic DNA, as recently published reports indicate.^32^,^33^,^41^,^42^ Finally, the supernatant specimen provides an additional option for testing molecular indicators of malignancy in spite of the possible presence of DNA from non-neoplastic cells. Consequently, the data presented here suggest that supernatant fluid should be regarded as a valuable source of information that may address many diagnostic issues and may serve as a useful, complimentary tool for pathologists when microscopic examination is suboptimal.
